# Framing orientation selectivity

**DOI:** 10.7554/eLife.39762

**Published:** 2018-08-14

**Authors:** Floris P de Lange, Matthias Ekman

**Affiliations:** 1 Donders InstituteRadboud UniversityNijmegenNetherlands

**Keywords:** Vignette, multivariate classification, primary visual cortex, fMRI, orientation column, V1, Human

## Abstract

The ongoing debate on the neural basis of orientation selectivity in the primary visual cortex continues.

**Related research article** Roth ZN, Heeger D, Merriam E. 2018. Stimulus vignetting and orientation selectivity in human visual cortex. *eLife* **7**:e37241. doi: 10.7554/eLife.37241

Color, contrast and motion are only some of the many things our brain needs to process when it receives information about our surroundings. From the moment light hits our eyes, the visual input is depicted and transported through a myriad of steps and networks.

In a region of the brain called the primary visual cortex or V1, the neurons are arranged in a specific way that allows the visual system to calculate where objects are in space. That is, neurons are organized ‘retinotopically’, meaning that neighboring areas in the retina correspond to neighboring areas in V1. Moreover, in humans, neurons sensitive to the same orientation are located in so-called orientation columns. For example, in one column, all neurons only respond to a horizontal stimulus, but not to diagonal or vertical ones (Figure 1A). Different orientation columns sit next to each other, repeating every 0.5–1 mm, and together cover the entire visual field.

**Figure 1.**
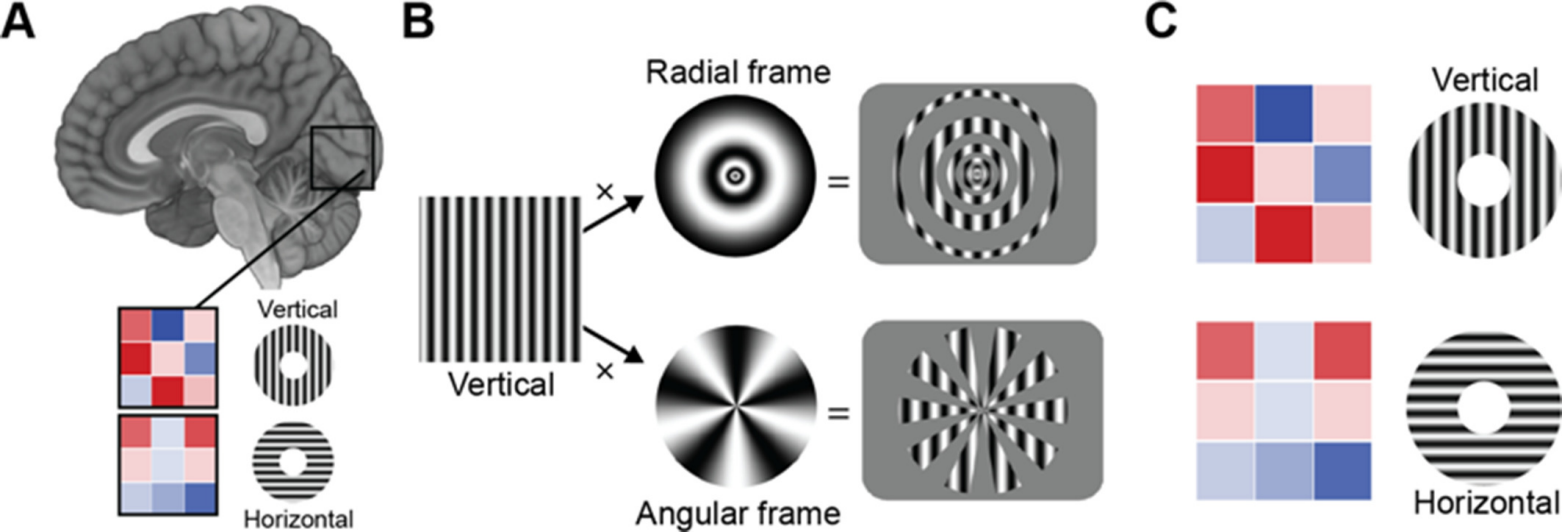
How can we discern orientation selectivity from fMRI measurements? (**A**) Differently oriented gratings (here vertical and horizontal) elicit different activity patterns in the primary visual cortex (illustrated by the 3x3 voxel matrix, where the color of each voxel is proportional to its activity (red is very active, blue is inactive). Multivariate pattern analysis techniques are used to decode the orientation of the grating from the voxel pattern. Roth et al. argue that variations in activity patterns are caused not by differences in the orientation of the stimuli per se, but are instead caused by ‘vignetting’ – a term they use to describe the interaction between the orientation of the and a change in light intensity that occurs for instance at the frame within which the stimulus is presented. (**B**) Applying different frames to the same vertical stimulus (left) (e.g., a radial frame (top), or an angular frame (bottom)) modulates the fMRI activity pattern in a way that is predicted by their computational model. (**C**) The same oriented grating can give rise to an opposite activity pattern, depending on whether it is projected through a radial frame (top) or angular frame (bottom).

Scientists often use a technique called functional magnetic resonance imaging, or fMRI for short, to study brain circuits. In 2005, two research groups managed to read out the orientation of a visual stimulus from fMRI activity patterns, a development that was met with a lot of excitement – but also some skepticism (Haynes and Rees, 2005; Kamitani and Tong, 2005). The resolution of fMRI is usually insufficient to image the orientation columns in V1: an ‘unbiased’ sample at the resolution of ~2–3 mm would capture neurons with all possible orientation preferences. How, then, was it possible to draw detailed conclusions from such a coarse-scale measure as fMRI?

Initially, it was speculated that even though every fMRI voxel contains all orientation columns (due to the much larger resolution of a voxel compared to the spatial scale of orientation columns), there are subtle differences between voxels in terms of the proportion of the different orientation columns within each voxel. For example, in one voxel, more horizontal than vertical columns may be found (Boynton, 2005). This is refered to as fine-scale bias. These subtle differences are random but systematic. Therefore, a machine learning algorithm can read out the orientation on the basis of these small differences.

Later studies suggested that, rather, the ability to decode orientation from fMRI patterns originates from activity differences at multiple spatial scales (Swisher et al., 2010), or even exclusively at a coarse spatial scale, i.e. at the level of retinotopic maps (Freeman et al., 2011). For example, clockwise orientation columns may be over-represented in neurons encoding the upper right part of our surrounding visual space, whereas counter-clockwise orientation columns may be over-represented in neurons encoding the upper left part of visual space (Sasaki et al., 2006). This is an example of coarse-scale bias. Now, in eLife, Zvi Roth, David Heeger and Elisha Merriam from the National Institutes of Health and the New York University add a new twist to this debate (Roth et al., 2018).

According to previous research, the edges of a visual stimulus (e.g., the outer and inner contours of a disc with horizontal or vertical stripes) create coarse-scale differences in visual activity (Carlson, 2014). These edges generate decodable activation patterns, and indeed stimuli with blurred edges are harder to decode than with sharper edges. Roth et al. show in an elegant combination of computational modeling and empirical fMRI work that orientation decoding is indeed sensitive to such ‘edge effects’. However, it does not depend on the edge per se, but on the interaction (which Roth et al. term ‘vignetting’) between the orientation of the stimulus and the frame within which the stimulus is presented (for example, a vertical pattern is presented on a circular frame, with a hole in the middle).

The researchers presented the stimuli within a radial or angular frame, to create different vignettes (Figure 1B). Their computational model predicted that a pattern oriented in the same way would create an opposite coarse-scale bias under these two different sets of vignettes. Indeed, their empirical data confirmed the model’s predictions: a vertical pattern within a radial frame showed an opposite bias to a vertical pattern within an angular frame, but the same bias as a horizontal pattern within an angular frame. This suggests that the different vignettes had a response pattern that was shifted by 90° (Figure 1C).

Does this have any implications for previous studies using ‘vignetted’ stimuli (e.g., Kamitani and Tong, 2005; Haynes and Rees, 2005)? Fortunately, the conclusions of these studies do not directly depend on the relative contribution of coarse-scale and fine-scale bias in activity patterns. However, the study by Roth et al. serves as a cautionary tale that multivariate pattern analyses – when used to identify activity patterns in the brain – have their limitation (Naselaris and Kay, 2015). Vignetting could produce activity patterns that resemble orientation tuning even in neurons that do not process orientation. This also applies to other techniques, such as electrophysiological recordings, if they use ‘vignetted’ stimuli.

While Roth et al. find no evidence for fine-scale biases, the strength of the correlation between the predicted and measured orientation preference is arguably modest and leaves room for other sources of orientation information. Some scientists argue that biases related to the frame within which stimuli are presented are not the sole contributor to orientation decoding in the visual cortex and that other sources of orientation selectivity might co-exist alongside vignetting (Wardle et al., 2017).

In conclusion, Roth et al. make a compelling case of how the frame in which a stimulus is presented can dramatically change the measured orientation preference, uncovering an important source of measured orientation information in brain recordings.
